# Profile of bioactive compounds in *Nymphaea alba* L. leaves growing in Egypt: hepatoprotective, antioxidant and anti-inflammatory activity

**DOI:** 10.1186/s12906-017-1561-2

**Published:** 2017-01-17

**Authors:** Riham Omar Bakr, Mona Mohamed El-Naa, Soumaya Saad Zaghloul, Mahmoud Mohamed Omar

**Affiliations:** 1 Pharmacognosy Department, Faculty of Pharmacy, October University for Modern Sciences and Arts (MSA), Giza, Egypt; 2 Pharmacology Department, Faculty of Pharmacy, October University for Modern Sciences and Arts (MSA), Giza, Egypt; 3 Pharmaceutics Department, Faculty of Pharmacy, October University for Modern Sciences and Arts (MSA), Giza, Egypt; 4 Axe of Regenerative Medicine, Faculty of Medicine, Universite Laval, Quebec City, Canada

**Keywords:** *Nymphaea alba*, Ellagitannins, Palmitic acid, Oxidative stress, Inflammation, Hepatotoxicity

## Abstract

**Background:**

*Nymphaea alba L*. represents an interesting field of study. Flowers have antioxidant and hepatoprotective effects, rhizomes constituents showed cytotoxic activity against liver cell carcinoma, while several *Nymphaea* species have been reported for their hepatoprotective effects. Leaves of *N. alba* have not been studied before. Therefore, in this study, in-depth characterization of the leaf phytoconstituents as well as its antioxidant and hepatoprotective activities have been performed where *N. alba* leaf extract was evaluated as a possible therapeutic alternative in hepatic disorders.

**Methods:**

The aqueous ethanolic extract (AEE, 70%) was investigated for its polyphenolic content identified by high-resolution electrospray ionisation mass spectrometry (HRESI-MS/MS), while the petroleum ether fraction was saponified, and the lipid profile was analysed using gas liquid chromatography (GLC) analysis and compared with reference standards. The hepatoprotective activity of two doses of the extract (100 and 200 mg/kg; P.O.) for 5 days was evaluated against CCl_4_-induced hepatotoxicity in male Wistar albino rats, in comparison with silymarin. Liver function tests; aspartate aminotransferase (AST), alanine aminotransferase (ALT), alkaline phosphatase (ALP), gamma glutamyl transpeptidase (GGT) and total bilirubin were performed. Oxidative stress parameters; malondialdehyde (MDA), reduced glutathione (GSH), catalase (CAT), superoxide dismutase (SOD), total antioxidant capacity (TAC) as well as inflammatory mediator; tumour necrosis factor (TNF)-α were detected in the liver homogenate. Histopathological examination of the liver and immunohistochemical staining of caspase-3 were performed

**Results:**

Fifty-three compounds were tentatively identified for the first time in *N.alba* leaf extract, where ellagitannins represent the main identified constituents. Nine hydrocarbons, two sterols and eleven fatty acids were identified in the petroleum ether extract where, palmitic acid and linolenic acids represented the major saturated and unsaturated fatty acid respectively. *N.alba* AEE significantly improved the liver function, oxidative stress parameters as well as TNF-α in addition to the amelioration of histopathological features of the liver and a profound decrease in caspase-3 expression.

**Conclusion:**

These results shed light on the hepatoprotective effect of *N. alba* that is comparable with that of silymarin. The antioxidant activities of *N. alba* extract in addition to the inhibition of crucial inflammatory mediator, as TNF-α, might be the possible hepatoprotective mechanisms.

## Background

Hepatotoxicity is a prevalent problem worldwide. Carbon tetrachloride (CCl_4_) is a chlorinated hydrocarbon that is commonly used in industries as a solvent and in medicine as a vermifuge. The compound is also found at low levels in ambient air and water [1]. Exposure to CCl_4_ is known to result in acute hepatotoxicity in humans and experimental animals. It is widely used in scientific research as a model of hepatotoxicity and to evaluate hepatoprotective agents [2, 3]. CCl_4_ is converted by cytochrome P450 2E1 to trichloromethyl free radical (CCl_3_∙) and trichloromethylperoxy radical (CCl_3_OO∙). Both radicals initiate lipid peroxidation and protein deterioration with subsequent damage of the cellular membrane and leakage of intracellular enzymes into the serum. These processes eventually lead to inactivation of the calcium pump with calcium influx and subsequent liver cell death. Moreover, lipid peroxidation and damage of hepatocyte membranes initiated by CCl_4_ was reported to be associated with the release of inflammatory mediators such as tumour necrosis factor (TNF)-α from activated hepatic macrophages, which potentiate CCl_4_-induced hepatic injury [3, 4].

*Nymphaea alba* L. (*N. alba*), known as the European water lily, White Lotus, or Nenuphar, is an aquatic flowering plant of the family Nymphaeaceae. *N. alba* was widely used in Indian folk medicinal products as an antiseptic, an astringent, radical scavenger, in burning and in insomnia while rhizomes are applied externally as a rubefacient [5]. Previously published studies reported the antioxidant, anti-inflammatory as well as hepatoprotective effect of *N. alba* flowers [6–8]. These effects may result from the phenolic constituents, including ellagic and gallic acid and their methyl and ethyl esters and flavonoids as aglycones of quercetin, kaempferol, isokaempferide, apigenin and their glycosides previously identified in the flowers [9, 10]. A recent study on the rhizomes revealed the presence of hydrolysable tannins, glycosylated phenolic acids and flavonoids. The methyl and ethyl gallate as well as pentagalloyl glucose, the main constituents identified, showed powerful cytotoxic activity against liver cell carcinoma [11]. Leaves of the white flowered water lily have been evaluated for their cytotoxic, antiproliferative and anxiolytic activities [12–14].

The broad range of traditional uses along with the previous reports concerning the hepatoprotective effect of *N. alba* flowers [7] as well as other *Nymphaea* species [15], and the absence of any reports about the phytochemical profile of *N. alba* leaf, aroused our interest in *N. alba* as a source of bioactive compounds. This study represents the first detailed chemical investigation of *N. alba* leaf that demonstrates the presence of a variety of free and conjugated forms of ellagic acid and ellagitannins tentatively identified by high-resolution electrospray ionisation mass spectrometry (HRESI-MS/MS) in the aqueous ethanolic extract. Hepatoprotective, antioxidant and anti-inflammatory activity of the *N. alba* leaf extract against CCl_4_- induced hepatotoxicity have also been studied and showed promising results.

## Methods

### Plant material

Leaves of *N. alba* L. were collected from El Orman Gardens, Giza, Egypt, in November, 2013 during the flowering stage. Authentication of the plant was performed by Dr. Therese Labib Youssef (consultant in plant taxonomy, Ministry of Agriculture). A voucher specimen (RS006) is deposited at the herbarium of the Pharmacognosy Department, Faculty of Pharmacy, October University for Modern Science and Arts, Egypt.

### Extraction

The powdered air-dried leaves of *N. alba* (300 g) were exhaustively extracted with aqueous ethanol (70% v/v) under reflux. After filtration, the aqueous ethanolic extract (AEE) was evaporated to dryness *in vacuum* at 40 °C to yield 33 g.

### Liquid chromatography coupled with High-resolution electrospray ionisation mass spectrometry (LC-HRESI-MS/MS)

LC-HRESI-MS/MS was performed on a Bruker Micro-TOF-Q Daltonics (API) Time-of-Flight mass spectrometer (Bremen, Germany), coupled to a 1200 series HPLC system (Agilent Technologies, Waldbronn, Germany), equipped with a high performance autosampler, binary pump, and PDA detector G 1314 C (SL). Chromatographic separation was performed on a Superspher 100 RP-18 (75 × 4 mm i.d.; 4 $\mu$m) column (Merck, Darmstadt, Germany).

### Identification of Phenolic Compounds of AEE of *N. alba* by LC-HRESI-MS/MS

The method was performed according to Hassaan et al. [16]. Injection volume was 10 $\mu$L. The solvents were: (A) 2% acetic acid (pH 2.6) and (B) 80% methanol, 2% acetic acid, and pH 2.6. The gradient elution was from 5 to 50% B at 30 °C at a flow rate of 100 $\mu$L/min. The ionization technique was an ion spray (pneumatically assisted electrospray). Spectra were recorded in positive and negative ion modes between *m/z* 120 and 1,500 with capillary voltage, 4000 V and heated dry nitrogen gas (temperature, 200 °C) and flow rate 10 L/min. The gas flow to the nebulizer was set at pressure 1.6 bar. For collision-induced dissociation (CID) MS/MS measurements, the voltage over the collision cell varied from 20 to 70 eV. Argon was used as the collision gas. Data analysis software was used for data interpretation. Sodium formate was used for calibration at the end of the LC/MS run. Interpretation for ESI-MS was performed by Xcalibur 2.2 SP1 software from Thermo Scientific (Berlin, Germany).

### Gas Liquid Chromatography (GLC) of Unsaponifiable Matter (USM) and Fatty Acid Methyl Ester (FAME)

Powdered air-dried leaves (100 g) were exhaustively extracted with petroleum ether (60–80 °C). The petroleum ether extract was filtered and evaporated under reduced pressure. The petroleum ether extract (1 g) was saponified by refluxing with ethanolic KOH (20%) at 60 °C for 2 h and then exhaustively extracted with ether. The combined ethereal extracts were washed, dehydrated over anhydrous sodium sulphate, evaporated to dryness and then analysed as unsaponifiable matter (USM) for the hydrocarbon and sterol contents. The saponified extract was acidified with HCl (5 N) and then extracted several times with ether. The combined ethereal extracts were evaporated to dryness, esterified into fatty acid methyl esters (FAMEs) by reflux with MeOH:H_2_SO_4_ (50:3) and extracted with ether [17].

The ether extracts of the USM and FAME fractions were analysed by GLC against the available authentic standards. Identification of hydrocarbons, sterols, and fatty acid methyl esters was carried out by comparing retention times of the peaks with those of the available authentic standards. FAMEs were analysed on a 70% Thermo Scientific Trace TR-FAME gas chromatographic (GC) capillary column packed with 70% Cyanopropyl Polysilphenylene-siloxane, 30 m x 0.25 mm id. The injector and detector temperatures were set at 250 and 300 °C, respectively. The temperature was increased 70 ° C to 190 °C at a rate of 8 °C /min. Nitrogen was used as carrier gas (30 ml/min).

USM was analysed on a Capillary HP6890 series, 1.5 m × 4 mm i.d. The injector and detector temperatures were set at 250 and 300 °C, respectively. The temperature was increased from 70 to 270 °C, at a rate of 10 °C /min. Nitrogen was used as the carrier gas (30 mL/min).

### 1, 1-Diphenyl-2-picrylhydrazyl (DPPH) radical scavenging activity

A weighed amount of AEE was dissolved in methanol (100 *μ*g/mL), screened for its free radical scavenging activity using the stable free radical DPPH, and then measured spectrophotometrically. The absorbance was measured at 517 nm and carried out in triplicate [18]. Radical scavenging activity was calculated by the following formula: DPPH scavenging effect (%) = [(A0 - A1)/A0) × 100], where A0 was the absorbance of the control reaction, and A1 was the absorbance of the sample [19]. The concentration of sample required to scavenge 50% of the DPPH was calculated from a graph plotted for the % inhibition against the concentration in *μ*g/mL. Ascorbic acid was used as standard.

### Hepatoprotective activity

#### Experimental animals

Eight-week-old male Wistar albino rats (200–220 g) were purchased from the National Institute of Ophthalmology, Egypt. The animals were kept in the animal house, October University for Modern Sciences and Arts (MSA), Egypt. All animals were kept in a pathogen-free facilities under standard laboratory conditions (temperature 25 ± 2 °C and 12 h light/12 h dark cycle) with free access to food and water. The animals were housed in groups of four in plastic cages with sawdust bedding. Experimental work was carried out in laboratories at MSA University, Egypt. Procedures involving animals and their care were in conformity with the institutional guidelines (Approval number of ethics committee, MSA University, EC 10 PG10/2011) and in compliance with national and international laws on the care and use of laboratory animals.

#### Experimental design

Two different doses of *N. alba* (100 and 200 mg/kg) were tested for their hepatoprotective effect against CCl_4_-induced hepatotoxicity. Doses and route of administration selection were according to previously published studies [12, 13]. Hepatotoxicity was induced by injection of a single intraperitoneal (I.P.) dose of CCl_4_ (0.5 ml/kg) [20].

A total of 40 rats were randomly divided into five groups ($n$ = 8). Group I (Control): received vehicles. Group II (CCl_4_): received CCl_4_ (0.5 ml/kg; I.P.) once. Group III (*N. alba* low dose): received CCl_4_ (0.5 ml/kg; I.P.) + *N. alba* extract (100 mg/kg; P.O.) 24 h after CCl_4_ for 5 days. Group IV (*N. alba* high dose): received CCl_4_ (0.5 ml/kg; I.P.) + *N. alba* (200 mg/kg; P.O.) 24 h after CCl_4_ for 5 days. Group V (Silymarin): received CCl_4_ (0.5 ml/kg; I.P.) + silymarin (100 mg/kg; P.O.) 24 h after CCl_4_ for 5 days. Treatments were given at 10 a.m. Twenty-four hours after the last dose of treatments, blood samples were collected from the retro-orbital plexus. Serum was separated by centrifugation and stored at −80 °C. Rats were sacrificed; livers were excised, rinsed in ice-cold saline and blotted dry. Slices of liver tissue were fixed in 10% neutral formalin for histopathological examination and immunostaining of caspase-3. The rest of the liver tissue was weighed and homogenized in phosphate buffer saline to prepare 10% homogenate and stored at −80 °C.

### Assessment of biochemical markers of hepatic injury

Biochemical parameters reflecting liver functions such as serum aspartate aminotransferase (AST), alanine aminotransferase (ALT), alkaline phosphatase (ALP), gamma glutamyl transpeptidase (GGT) and total bilirubin were estimated using commercially available kits, according to the manufacturer instructions (Spectrum, Egypt).

### Assessment of oxidative stress in the liver

Liver malondialdehyde (MDA) and reduced glutathione (GSH) contents, catalase (CAT) and superoxide dismutase (SOD) activities and total antioxidant capacity (TAC) were assessed spectrophotometrically using commercial kits supplied by Bio-diagnostic (Bio-diagnostic, Egypt).

### Estimation of inflammatory cytokine, TNF-α

TNF-α content was measured in liver homogenate using an ELISA kit (BioLegend ELISA MAX™ Deluxe kit; BioLegend, San Diego, CA, USA). The assay was performed according to the manufacturer’s protocol.

### Histopathological examination of the liver

Liver specimens in 10% neutral formalin were embedded in paraffin and cut into 4 μm thick sections. Sections were stained with haematoxylin and eosin (H&E) and examined under a light microscope for histological changes.

### Immunohistochemical staining for caspase-3 in liver

Caspase-3 expression in the liver was detected by immunostaining of sections prepared from formalin-fixed, paraffin-embedded livers using caspase-3 detection kits according to the manufacturer instructions. The intensity of caspase-3 immunostaining was assessed as follows: 0 – none, 1 – mild, 2 – moderate and 3 – strong. The Immunohistochemical histological score (H-score) was calculated by multiplying the intensity by the percentage of caspase-3 positive cells, creating a range of possible scores of 0–300 [21].

### Statistical analysis

Data from animal work are expressed as the mean ± standard error of the mean (SEM). Comparisons between different groups were carried out by one-way analysis of variance (ANOVA) followed by the Tukey-Kramer test. The level of significance was set at *p* < 0.05. Graphpad software instat (version 2) was used to carry out statistical analysis.

## Results

### Phytochemical investigation

#### HRESI-MS/MS analysis of *N. alba* AEE

The chemical constituents in *N. alba* AEE were identified and characterized in both negative and positive ESI modes. The retention times and fragmentation patterns of the identified compounds are listed in Table 1. Compounds were tentatively identified based on matching their masses and fragmentation pattern with the literature information and ChemSpider. MS fragmentation interpretation is not discussed except when of special interest.

**Table 1:** Peak assignments and tentative identification of the major constituents in *N. alba* AEE by HRESI-MS/MS^a^ in the positive and negative modes

Peak Number	Tentatively Identified Compound	tR^b^ (min.)	[M-H]^−^m/z^d^	Negative Ionization MS/MS^c^	[M + H]^+^m/z^d^	Positive Ionization MS/MS
1	HHDP^e^ -hexoside	1.85	481.06	**301.14** 463.2 275.18	483.08	**465.14** 437.07 309.07 263
2	Epicatechin derivative	2.94	427.09	**265.15** 307.14 367.23 289.19 247.1 221.15		
3	Ellagitannin derivative	3.59	817.07	**773** 481 301.11 481.24 275.19 247.23	819.09	**801.2** 481.13 463.05 337.14 319.14 303.12
4	HHDP-galloyl-ellagic acid	4.12	773.09	**301.17** 729 471.27 275.24 247		
5	Ellagic acid	4.44	301.15	**257.22** 229.10 185.16	303.1	**275.03** 257.1 247.04 229.13 165.02
6	Isorhamnetin derivative	5.38	386.96	**315** 343.21 271.16 255.16 189.02 161.06		
7	Quercetin 3–*O*-acetyl hexoside	12.73	505.07	**459.19** 403.18 487.14 301.12 275.17 231.25 247.09 169.12	507.09	**489.14** 447.14 405.14 387.03 303.18 205.14187.17
8	Ellagic rhamnosyl hexoside	13.27	609.17	**403.17** 563.18 447.3 429.2 359.2 291.22 301.13 275.28 247.19	611.18	**593.30** 551.30 533.27 449.30 389.21 343.21 303.22
9	Lagerstannin A (Bis-HHDP-gluconic acid)	13.40	799.06	**497.16** 301.21 755.24 453.26	801.08	**463.15** 765.11 783.17 481.15 337.12 303.15 277.10 247.15
10	Brevifolin	13.5	247.02	**203.11** bp 175.13	249.6	**207.05** 232.12 221.12 187.08 159.04 131.04
11	Phyllanthusiin U	13.72	924.11	**301.01** 622.2 604.24 905.22 451.19	926.13	**908.2** 303.12 277.15 703.25 606.26
12	Valoneic acid dilactone dimer	13.81	939.02	**469.24** 425.21 300.2	941.02	**453.15** 922.27 621.2 470.99 407.19
13	3,4,8,9,10-Pentahydroxydibenzo[b,d]pyran-6-one	14.13	275.02	**245.05** 257.08 231.1 229.2 203.08 187.18	277.03	**235.23** 221.17 183.17
14	Luteolin	14.34	285.04	**257.16** 241.13 229.2 213.23 185.2 167.24		
15	Ellagitannin derivative	14.45	931.11	**301.14** 783.2	933.03	**873.05** 915
16	Methyl gallate	14.56	183.03	**168.13** 124.03 169.09	185.33	**167.07** 153.03 104.96 129.07
17	Ellagic acid hexoside	15.54	463.05	**301.2** 419.2 417.16 331.7 274.96 251.07 247.2	465.05	**303.09** 447.13
18	Catechin or epicatechin derivative	15.83	621.07	**575.46** 439.15 289.19 245.10 217.14		
19	Orientin	16.3	447.02	**403.16** 233.03 427.03 359.17 277.12 357.18 329.16 327.18 315.23 301.19 287.17 189.05		
20	Ellagitannin derivative	16.63	755.07	**711.24** 453.19 301.21 435.21 409.36 275 247.23		
21	Phyllanthusiin E	17.04	291.04	**247.11** 203.18	293.03	**247.11** 275.06
22	Ellagitannin derivative	17.17	1153.09	**799.17** 755.21 453.16 409.24		
23	Phyllanthusiin B isomer	18.05	969.09	**631.25** 667.22 451.15 331.27 291.07		
24	Castalin derivative	18.77	967.07	**631.14** 451.2 571.19	969.08	**950.82** 499.10 579.02 481.16 453.10 355.1 337.17
25	HHDP-gluconic acid	19.21	497.06	**463.20** 453.24 301.07 291.18 275.18 257.25 247.23		
26	Ellagic acid-galloyl hexoside	20.85	613.05	**451.13** 425.26 569.22 509.16		
27	Phyllanthusiin B	21.17	969.09	**633.26** 923.18 951.26 667.26 ch301.24 451.30 275.18	971.1	**953.16** 651.14 633.17 615.15 481.21 453.18 303.15 277.17
28	Vescalagin or castalagin	21.28	933.06	**613.20** 301.15 631.2 273.14 915.21	935.07	**916.31** 615.34 481.12 453.13 303.19 277.18
29	Apigenin	24.66	269.03	**223.01** 179.07		
30	Phyllanthusiin C	24.91	925.10	**605.29** 301.08 623.23 905.15 633.08 453.09 291.18 273.06	927.11	**909.26** 607.23 589.14 571.16 419.23 321.19 303.1 277.11
31	Corilagin (Galloyl HHDP hexoside)	25.14	633.07	**301.14** 589.22 481.29 275.27 257.15	635.08	**443.11** 617.23 573.16 355.11 254.15 191.12
32	Pedunculagin (Bis HHDP-hexoside)	25.77	783.07	**481.17** 737.33 301.11 451.11	785.08	**767.15**
33	Castalin	26.17	631.06	**301.20** 299.12 451.18 271.05	633.07	**615.12**
34	Phyllanthusiin C derivative	27.24	1217.12	**925.56**		
35	Lagerstannin C (Galloyl-HHDP-gluconic acid	27.33	649.1	**603.26** 605.27 631.23 469.18 441.17 451.26 301.14 247.19	651.08	**633.13** 481.19 337.11
36	Catechin or epicatechin	28.29	289.12	**245.09** 217.18	291.42	**247.1** 272.03 235.09 219.07 191.07
37	Lagerstannin B (flavogalloyl HHDP-gluconic acid)	29.82	949.11	**647.25** 905.25 629.24 477.24 333.21 301.15 275.2		
38	Granatin B(Galloyl-HHDP-DHHDP-hex)	30.97	951.08	**605.26** 649.21 301.15 631.23 907.21 952.19 497.23	953.08	**277.10** 935.15 633.24 615.12 321.11 303.11
39	Dehydrated tergallic C-glucoside	31.4	613.05	**299.07** 301.27 569.27 595.15 227.15	615.06	**453.14** 387.09 313.15
40	Cinnamic acid derv.	31.5	329.09	**197.10** 239.1 169.07	331.13	**299.09** 313.19 272.12 236.09 151.04
41	Phyllanthusin B derivative	32.18	981.59	**969.23** 933.23 613.24 481.26		
42	Valoneic acid dilactone	32.47	469	**425.15** 301.09		
43	Gallic acid derivative	32.61	198.62	**169.06** 153.14 125.21 117.06		
44	Ellagitannin derivative	33.31	907.08	**605.19** 863.09 587..23 561.23 301.14 275.11	909.09	**890.22** 703.08 589.21 321.19 303.05 277.09
45	Digalloyl ellagic acid	33.62	605.62	**561.25** 247.18 291.12 203.18 453.24 435.12 229.08	607	**315.05** 589.27 565.23 549.25 505.27
46	Castalgin derivative	34.51	965.09	**631.22** 301.03	967.10	**647.25** 949.1 906.2 629.26 495.16 445.26 303.18 27.10
47	Chebulagic acid	34.79	953.18	**935.32**, 907.22 649.28 605.1 631.28 301.22 291.24	955.19	**937.20** 633.17 481.26 463.28 319.11 301.16 27.19
48	Ellagic acid pentoside	38.56	433.04	291.15 bp 405.17 301.18 275.23 247 229	435.15	417.19 bp 407.25 399.14 376.35 343.11 325.11 299.19 181.19
49	Ellagic acid rhamnosyl	39.07	447.02	**359.17** 403.2 385.11 315.25 301.13 275.18	448.98	**413.09** 405.24 331.08 277.1
50	Geraniin	40.40	951.07	**933.10** 613.20 301.13 631.15		
51	Vescalagin derivative	40.8	965.09	**613.2** 933.19 301.09	967.10	**935.17** 647.22 277.12
52	Galloyl ellagic acid	41.83	453.04	301.13 438.34 291.12 247.11 169.13 273.27	455.08	**437.13** 394.98
53	Gallic acid	62.11	169.01	**125.11**		

Boldface digits reflects the base peak (100% abundance)

Fragment ions are listed in order of relative abundances

^a^High resolution liquid chromatography coupled with electrospray ionisation mass /mass spectometry

^b^Retention time

^c^Mass/Mass spectrometry

^d^Mass to charge ratio

^e^Hexahydroxydiphenic acid

Table 1 outlines 53 compounds, among them 42 hydrolysable tannins, three simple phenolic acids, and eight flavonoids were detected.

#### Estimation of USM and FAME

Nine hydrocarbons, two sterols and eleven fatty acids were identified in *N. alba* AEE. The percentage content of individual hydrocarbons and fatty acids are summarized in Tables 2 and 3. The percentage of identified hydrocarbons was estimated as 94.9% while the sterol content represented 5.03%*. n*-Tetracosane was determined as the major hydrocarbon (59.6%), *n*-tetratriacontane was detected as the second most abundant hydrocarbon (18.96%) while β-sitosterol was of significant percentage (3.5%). By comparison with the FAME standards, the percentage of the saturated fatty acid represents 49.3% where the major saturated fatty acid was palmitic acid (40.8%) while the major unsaturated fatty acids were linolenic acid (24.5%), linoleic acid (16.8%) and palmitoleic acid (8.5%).

**Table 2:** Unsaponifiable content in *N. alba*

Compound	Percent
*n*-tetradecane, C14	0.97
*n*-pentadecane, C15	0.84
*n*- hexadecane, C16	1.11
*n*-octadecane, C18	9.69
*n*-nonadecane, C19	1.13
*n*-tetracosane, C24	59.59
*n*-octacosane, C28	2.67
*n*-tetratriacontane, C30	18.96
Total hydrocarbons	94.96
stigmasterol	1.68
β-sitosterol	3.35
Total sterols	5.03
% Unidentified	0.006

**Table 3:** Fatty acid composition (%) of *N. alba*

Compound	Percent
Octanoic (Caprylic) acid, C8:0	1.22
Decanoic (Capric) acid, C10:0	1.67
Tetradecanoic (Myristic) acid, C14:0	1.64
Hexadecanoic (Palmitic) acid, C16:0	40.84
Octadecanoic (Stearic) acid, C18	1.43
Eicosanoic (Archidic) acid, C20	2.51
∑SFA^a^	49.31
cis-9-Hexadecanoic(Palmitoleic) acid, C16:1ω 7	8.41
cis-9-Octadecanoic (Oleic) acid, C18:1ω9	1.04
∑MUFA^b^	9.45
cis,cis-9,12-Octadecadienoic (Linolenic) acid, C18:3, ω6	24.45
All cis-6,12,15-Octadecotrionic (Linoleic) acid, C18:2 ω6	16.78
∑PUFAs^c^	41.23
Total unsaturation	50.68

^a^Saturated fatty acids

^b^Monounsaturated fatty acids

^c^Polyunsaturated fatty acids

### 1, 1-Diphenyl-2-picrylhydrazyl (DPPH) radical scavenging activity

AEE showed strong DPPH scavenging activity as indicated by low IC_50_ (5.2 ± 0.3 *μ*g/mL) and LC_90_ (9.1 ± 0.27 *μ*g/mL) compared with ascorbic acid (12 ± 3.5 *μ*g/mL).

### Hepatoprotective activity

#### Effect of *N. alba* on liver function parameters

Serum level of liver functions parameters; ALT, AST, GGT, ALP and total bilirubin were significantly increased in CCl_4_-intoxicated rats compared with normal level (*P* < 0.05; Table 4). Treatment with *N. alba* (100 and 200 mg/kg) resulted in significant decrease of ALT, AST, GGT, ALP and total bilirubin compared with CCl_4_-intoxicated rats in a dose dependant manner (*P* < 0.05; Table 4). Similarly, silymarin significantly improved the liver function parameters compared with CCl_4_ group (*P* < 0.05; Table 4).

**Table 4:** Effect of *N.alba* AEE on CCl_4_-induced changes in the liver function parameters in rats. Rats were intoxicated with CCl_4_ (0.5 ml/kg; I.P.) and treated with *N. alba* (100 and 200 mg/kg; P.O.) and silymarin (100 mg/kg; P.O.) for 5 days. ALT, AST, total bilirubin, GGT and ALP were measured

Parameter	Control	CCl_4_	*N.alba* extract		Silymarin (100 mg/kg)
			Low Dose (100 mg/kg)	High Dose (200 mg/kg)	
ALT (U/L)^1^	32.6 ± 3.1	88.1 ± 7.3^a^	58.4 ± 4^b^	41.9 ± 2.6^b^	38.1 ± 2.2^b^
AST (U/L)^2^	68.5 ± 3.5	139 ± 8.3^a^	105.9 ± 4.1^b^	82.1 ± 6.1^b^	72.5 ± 4.7^b^
Total Bilirubin (mg/dl)	0.2 ± 0.02	1.2 ± 0.1^a^	0.5 ± 0.03^b^	0.3 ± 0.03^b^	0.2 ± 0.02^b^
GGT (U/L)^3^	7.7 ± 0.6	35.9 ± 3.3^a^	20.8 ± 1.8^b^	12.9 ± 1.2^b^	12 ± 1.2^b^
ALP (U/L)^4^	278.5 ± 19.1	496.5 ± 38.3^a^	414.7 ± 10.9^b^	346.6 ± 22.8^b^	294.2 ± 15.4^b^

Data are presented as the mean ± SEM, *n* = 8. ^a^Significant difference from control group; *P* < 0.05. ^b^Significant difference from CCl_4_ group; *P* < 0.05

^1^Alanine aminotransferase

^2^Aspartate aminotransferase

^3^Gamma glutamyl transpeptidase

^4^Alkaline phosphatase

#### Effect of *N. alba* on oxidant status of the liver

**Fig. 1:**
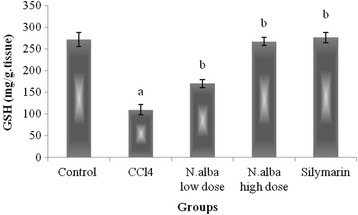
The effect of *N. alba* AEE on the liver content of GSH in CCl_4_-intoxicated rats. Rats were intoxicated with CCl_4_ (0.5 ml/kg; I.P.) and treated with *N. alba* (100 and 200 mg/kg; P.O.) and silymarin (100 mg/kg; P.O.) for 5 days. GSH was determined in the liver homogenate. Data are presented as the mean ± SEM, *n* = 8. ^a^Significant difference from control group; *P* < 0.05. ^b^Significant difference from CCl_4_ group; *P* < 0.05

**Fig. 2:**
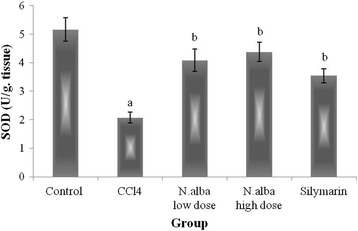
The effect of *N. alba* AEE on the liver SOD activity in CCl_4_-intoxicated rats. Rats were intoxicated with CCl_4_ (0.5 ml/kg; I.P.) and treated with *N. alba* (100 and 200 mg/kg; P.O.) and silymarin (100 mg/kg; P.O.) for 5 days. SOD was determined in the liver homogenate. Data are presented as the mean ± SEM, *n* = 8. ^a^Significant difference from control group; *P* < 0.05. ^b^Significant difference from CCl_4_ group; *P* < 0.05

**Fig. 3:**
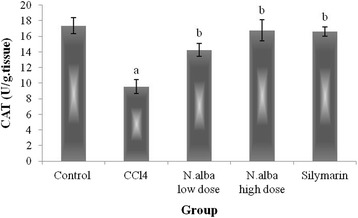
The effect of *N. alba* AEE on the liver CAT activity in CCl_4_-intoxicated rats. Rats were intoxicated with CCl_4_ (0.5 ml/kg; I.P.) and treated with *N. alba* (100 and 200 mg/kg; P.O.) and silymarin (100 mg/kg; P.O.) for 5 days. CAT was determined in the liver homogenate. Data are presented as the mean ± SEM, *n* = 8. ^a^Significant difference from control group; *P* < 0.05. ^b^Significant difference from CCl_4_ group; *P* < 0.05

**Fig. 4:**
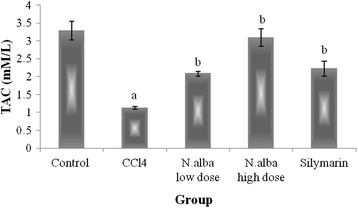
The effect of *N. alba* AEE on the liver TAC in CCl_4_-intoxicated rats. Rats were intoxicated with CCl_4_ (0.5 ml/kg; I.P.) and treated with *N. alba* (100 and 200 mg/kg; P.O.) and silymarin (100 mg/kg; P.O.) for 5 days. TAC was determined in the liver homogenate. Data are presented as the mean ± SEM, *n* = 8. ^a^Significant difference from control group; *P* < 0.05. ^b^Significant difference from CCl_4_ group; *P* < 0.05

**Fig. 5:**
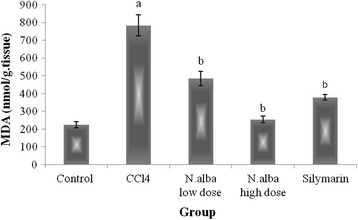
The effect of *N. alba* AEE on the liver content of MDA in CCl_4_-intoxicated rats. Rats were intoxicated with CCl_4_ (0.5 ml/kg; I.P.) and treated with *N. alba* (100 and 200 mg/kg; P.O.) and silymarin (100 mg/kg; P.O.) for 5 days. MDA was determined in the liver homogenate. Data are presented as the mean ± SEM, *n* = 8. ^a^Significant difference from control group; *P* < 0.05. ^b^Significant difference from CCl_4_ group; *P* < 0.05

Injection of CCl_4_ resulted in depletion of hepatic GSH content (59.7%), decrease in the activities of SOD and CAT (59.9 and 44.9%, respectively) and decline in TAC of the liver (65.7%) (*P* < 0.05; Figs. 1, 2, 3 and 4, respectively) compared with control group. Also, CCl_4_ significantly increased the liver lipid peroxidation product, MDA (249.1%) (*P* < 0.05; Fig. 5) compared with control group.

Treatment of CCl_4_-intoxicated rats with *N. alba* (100 and 200 mg/kg) significantly increased the liver content of GSH (55.1 and 143.4%, respectively; *P* < 0.05; Fig. 1) compared with the CCl_4_ group. *N. alba* (100 and 200 mg/kg) significantly enhanced the enzymatic activities of both SOD (79.1 and 111.1%, respectively) and CAT (49.2 and 75.5%, respectively) (*P* < 0.05; Figs. 2 and 3, respectively) compared with the CCl_4_ group. Furthermore, TAC of the liver increased significantly (84.1 and 173.5%) by treatment with the *N. alba* (100 and 200 mg/kg, respectively) (*P* < 0.05; Fig. 4). On the other hand, treatment with *N. alba* (100 and 200 mg/kg) significantly decreased liver MDA content (38.2 and 67.6%), respectively, compared with the CCl_4_ group (*P* < 0.05; Fig. 5).

Silymarin significantly increased the liver GSH content and TAC (151.7 and 97.3%, respectively) compared with the CCl_4_ group (*P* < 0.05; Figs. 1 and 4, respectively). The activities of SOD and CAT improved significantly (71 and 73.8%, respectively) by treatment with silymarin compared with the CCl_4_ group (*P* < 0.05; Figs. 2 and 3, respectively). The hepatic content of MDA decreased significantly (51.8.7%) compared with the CCl_4_ group (*P* < 0.05; Fig. 5).

#### Effect of *N. alba* on hepatic content of TNF-α

**Fig. 6:**
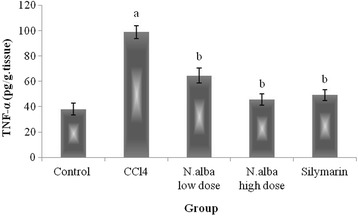
The effect of *N. alba* AEE on the liver content of TNF-α in CCl_4_-intoxicated rats. Rats were intoxicated with CCl_4_ (0.5 ml/kg; I.P.) and treated with *N. alba* (100 and 200 mg/kg; P.O.) and silymarin (100 mg/kg; P.O.) for 5 days. TNF-α was determined in the liver homogenate. Data are presented as the mean ± SEM, *n* = 8. ^a^Significant difference from control group; *P* < 0.05. ^b^Significant difference from CCl_4_ group; *P* < 0.05

Hepatic content of TNF-α was significantly increased in CCl_4_ group compared with control rats. Treatment with *N. alba* (100 and 200 mg/kg) resulted in significant decrease of TNF-α in a dose dependant manner (Fig. 6). Silymarin significantly improved the hepatic content of TNF-α compared with CCl_4_ control group (Fig. 6).

### Histopathological examination of hepatic tissue

**Fig. 7:**
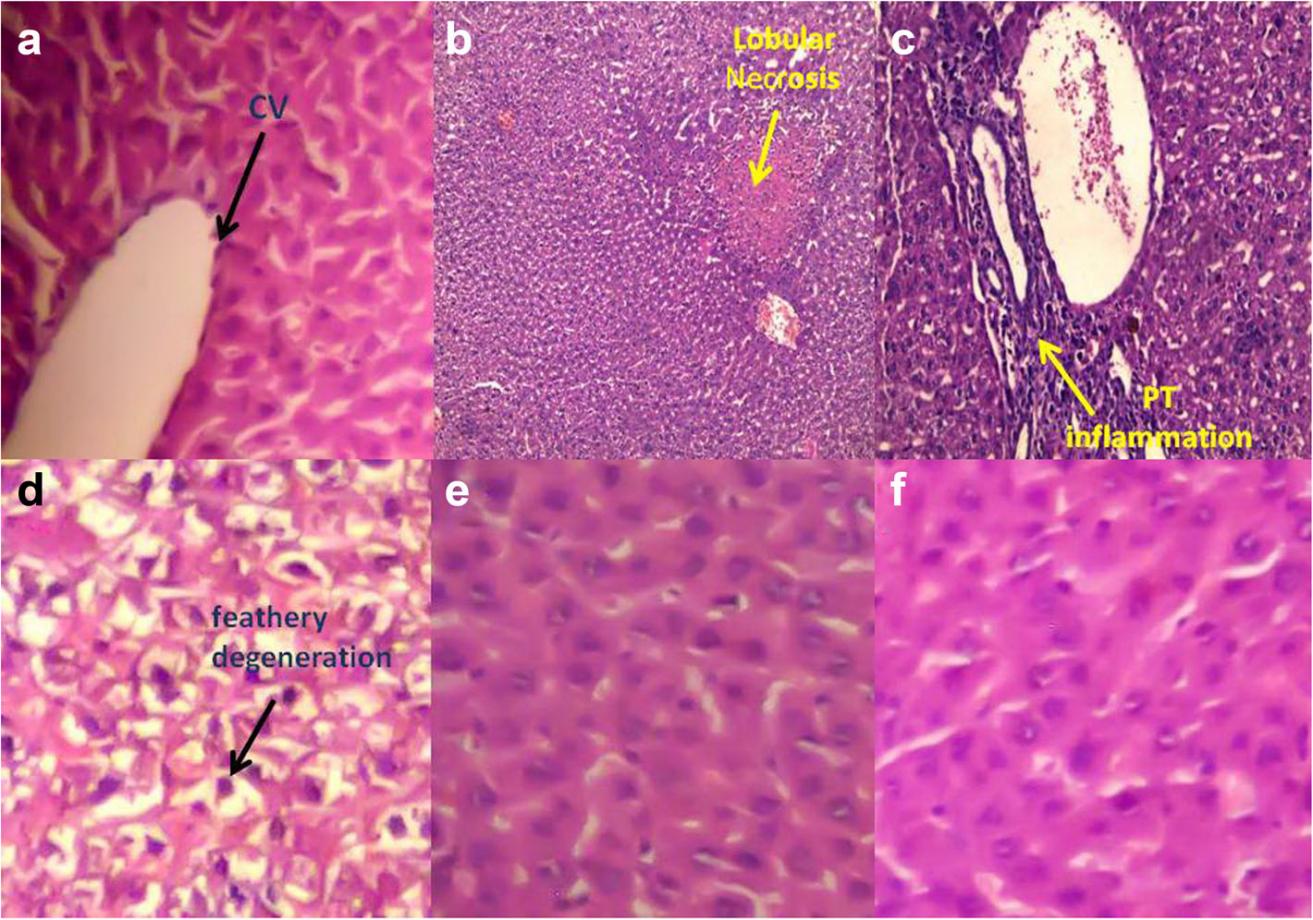
Representative photomicrographs of histopathological examination of the liver. **a** Liver of control rats (**b** and **c**) Liver of rats intoxicated with CCl_4_ (0.5 ml/kg; P.O.) showing severe feathery degeneration of hepatocytes and lobular necrosis (B) and portal lymphocytic infiltration (**c**). **d** Liver of rats intoxicated with CCl_4_ and treated with *N. alba* (100 mg/kg; P.O.) showing slight improvement of feathery degeneration of hepatocytes. **e** Liver of rats intoxicated with CCl_4_ and treated with *N. alba* (200 mg/kg; P.O.) showed marked improvement of the histopathological features. **f** Liver of rats intoxicated with CCl_4_ and treated with silymarin (100 mg/kg; P.O.)

Livers excised from a control group showed a normal architecture of hepatocyte lobules with normal central and portal areas and normal hepatocytes (Fig. 7a). The liver samples from CCl_4_-intoxicated rats showed severe feathery degeneration and necrosis of hepatocytes (Fig. 7b). The portal area showed severe infiltration by lymphocytes and dilation of the central vein (Fig. 7c). Rats treated with low dose of *N. alba* (100 mg/kg; P.O.) for 5 days showed slight improvement of the histopathological features of the liver compared with the CCl_4_-intoxicated group (Fig. 7d). However, a high dose of *N. alba* (200 mg/kg; P.O.) and silymarin showed marked advances in the liver features and disappearance of the feathery degeneration of hepatocytes (Fig. 7e and f, respectively).

### Immunohistochemical staining of caspase-3

**Fig. 8:**
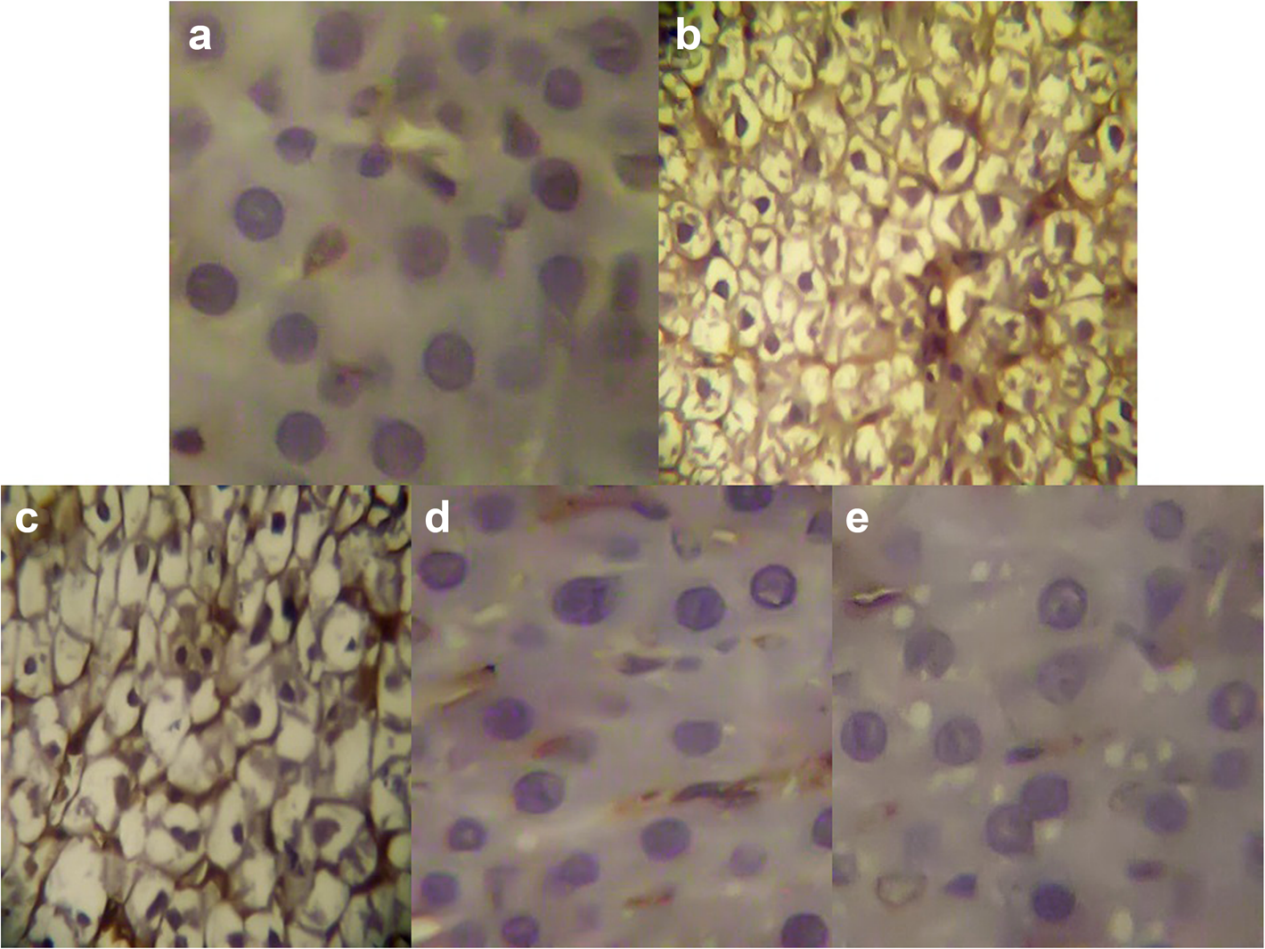
Representative photomicrographs of immunohistochemical staining of caspase-3. **a** Liver of control rats, (**b**) Liver of rats intoxicated with CCl_4_ (0.5 ml/kg; P.O.), (**c**) Liver of rats intoxicated with CCl_4_ and treated with *N. alba* (100 mg/kg; P.O.), (**d**) Liver of rats intoxicated with CCl_4_ and treated with *N. alba* (200 mg/kg; P.O.), and (**e**) Liver of rats intoxicated with CCl_4_ and treated with silymarin (100 mg/kg; P.O.)

Liver excised from rats injected with CCl_4_ showed high caspase-3 expression (H score = 60; Fig. 8b) while, caspase-3 was negatively stained in the control group (H score = 0; Fig. 8a). Caspase-3 expression was decreased in CCl_4_-intoxicated rats treated with low dose of *N. alba* (100 mg/kg; H score = 20; Fig. 8c). CCl_4_-intoxicated rats received the high dose of *N. alba* as well as those received silymarin (Figs. 8d and e, respectively) showed negative staining for caspase-3 (H score = 0).

## Discussion

The hyphenated HPLC-MS technique is an important method used for identifying complex mixtures, especially the phenolics in the crude extracts or the fraction found in the plant, either by using standard compounds (cochromatography) or by comparing mass spectra obtained with the literature (tentative identification) [22]. A chemical characteristic of the order Nymphaeals, which includes the family Nymphaeaceae, is the occurrence of significant amounts of gallic acid and ellagic acid [23]. Ellagitannins attracted considerable attention because of their vast structural diversity and biological activity, including antioxidant, antiviral and antitumor activity [24, 25].

*N. alba* flowers and rhizomes are known for their high phenolic content [9–11], while leaves have never been well studied before. In this study, *N. alba* AEE was demonstrated as a very rich source of phenolic compounds where, hydrolysable tannins were the main polyphenols identified (forty-two compounds) distinguished by their characteristic fragment ion spectra yielding losses of galloyl (m/z 152), gallate (m/z 170) and esters of hexahydroxydiphenic acid (HHDP) residues (m/z 302), while the common loss of 44 amu indicates the presence of a free carboxylic group (COOH) [26, 27].

A total of twenty-six ellagitannins were tentatively identified. HHDP and a polyol and in some cases, gallic acid represent the majority of *Nymphaea* constituents. Peak **1** with a precursor ion at *m/z* 481 was identified as HHDP-glucose [28, 29]. The presence of HHDP was supported by the formation of *m/z* 301 in the negative ionization and 303 in the positive.

Peak **31**, shows *m/z* at 633 [M-H] and fragment ions at *m/z* 301 [M-H-152-180], 589 [M-H-44], 481 [M-H-152] was tentatively identified as a galloyl-HHDP-glucose (corilagin) isomer, agreeing with Fischer et al., [30] and Barros et al. [31]. Peak **32** shows *m/z* at 783 [M-H] and fragments at *m/z* 481 [M-H-302] and 301 [M-H-302-180] was identified as pedunculagin (Bis HHDP hexoside), the release of one HHDP molecule yielded 481 (peak 1) [32]. Two compounds with *m/z* 951 were detected at different retention time (Peak **38** and **50**) which significantly differ in their fragmentation pattern, indicating the presence of isomeric structures which is common with ellagitannins. They were tentatively identified as granatin B and geraniin respectively [33, 34].

Three lagerstannins previously identified in *Lagerstroemia speciosa* were tentatively identified with the presence of gluconic acid. The common loss of 44 amu indicates the presence of a free carboxylic group [35]. Peak **35** shows *m/z* at 649 [M-H] and fragments at *m/z* 603 [M-H-44], 631 [M-H-18], 469 [M-H-180], 451 [M-H-44-152] and 301, which was identified as lagerstannin C (galloyl HHDP-gluconic acid). Peak **9** presents *m/z* at 799 [M-H] and fragments at *m/z* 755 [M-H-44], 497 [M-H-302] and 301 [M-H-302-196] and identified as lagerstannin A (Bis-HHDP-gluconic acid). Peak **37** shows *m/z* at 949 [M-H] and fragments at *m/z* 647 [M-H-302], 905 [M-H-44], 629 [M-H-44-152], 477 [M-H-302-170], 301 [M-H-302-170-176]. This peak was tentatively identified as Lagerstannin B (flavogalloyl-HHDP-gluconic acid) [30, 35].

Analysis of peaks **11**, **27**, 30 and **47**, yielded intense product ions resulting from the loss of the HHDP and/or galloyl moiety. These peaks were tentatively identified as phyllanthusiin U, B, C and chebulagic acid detected with [M-H] at *m/z* 924, 969, 925 and 953, respectively and previously identified in *Phyllanthus urinaria* [36, 37]. Peak **28** with a pseudomolecular ion [M-H] at *m/z* 933.06 and fragment ions at *m/z* 915, 631, 451 and 301 were in agreement with the fragmentation pattern attributed to castalagin [38]. The release of one HHDP or ellagic acid moiety (302 Da) from castalagin generated Peak **33** with an [M-H] at *m/z* 631, showing the typical ellagic acid fragments at m/z 299 and m/z 271 and tentatively identified as castalin [28, 30].

The free ellagic acid was confirmed by its MS data and MS/MS fragmentation (peak **5**), having *m/z* at 301 [M-H] in the negative mode and fragment ions at *m/z* 275, 257, 247, 229 and 185 [38]. Peaks **17**, **48**, **49** were tentatively identified as the glycosylated forms of ellagic acid with [M-H] at *m/z* 463, 433 and 447, respectively, showing the characteristic fragments of ellagic acid at *m/z* 301 and 275 in addition to the characteristic losses of a hexosyl, pentosyl and rhamnosyl residue, so, identified as ellagic acid hexoside, pentoside and rhamnoside respectively [30]. Peak **42** presents *m/z* at 469 [M-H] and a fragment at *m/z* 425, was tentatively identified as valoneic acid dilactone, a compound that often occurs in plants containing ellagitannins [39]. While peak **12** represented a dimer of valoneic acid dilactone with [M-H] at *m/z* 939 and main fragments at 469 and 425.

Gallic acid and its derivatives were also tentatively identified where gallic acid appeared at peak **53** while peak **39** was tentatively identified as dehydrated tergallic acid with a pseudomolecular ion [M-H] at *m/z* 613 and fragment ions at *m/z* 569 [M-H-44], 461 [M-H-152] and 299 [M-H-152-162] [29, 30].

Flavonoids have also been detected where Peak **8** shows *m/z* at 505 [M-H] and daughter ion at *m/z* 301 [M-acetyl hexoside], was tentatively identified as quercetin 3-*O*-acetyl hexoside that was previously identified in the *Nymphaea* species [40]. Peak **31** has an [M-H] ion at *m/z* 289 and base peak at *m/z* 245 [M-H-44] was tentatively identified as catechin or epicatechin by Pérez-Magariño et al., [41].

Beside the phenolic content *N. alba* extract appeared also as rich source of fatty acid. Essential fatty acids (EFAs) such as linolenic, linoleic and oleic acids help to raise HDL cholesterol, supporting cardiovascular, reproductive and immune systems. *N. alba* extract contains several essential fatty acids as linoleic (16.78%) and linolenic acid (24.45%), and, therefore, has a potential nutritional value in agreement with Eromosele and Eromosele, [42]. In addition, *N. alba* provided a rich source of β-sitosterol (5%), which is reported to reverse the impairment of the glutathione/oxidized glutathione ratio induced by phorbol esters in macrophage cultures with the increase in manganese superoxide dismutase and glutathione peroxidase activities and the decrease in catalase activity [43].

Oxidative stress plays a crucial role in the development of the aging process and some chronic diseases [21]. The antioxidant potential of medicinal plants is attributed to the redox properties of the phenolic compounds and there are several reports that correlate the total phenolic content to the antioxidant activity [44–46]. *N. alba* was shown as a potent radical scavenger with low IC_50_ (5.2 ± 0.3 *μ*g/mL) compared with ascorbic acid. This high radical scavenging activity suggests the ability of *N. alba* to reduce oxidative stress.

In this study, the hepatoprotective effect of *N. alba* AEE against CCl_4_-induced hepatotoxicity was demonstrated for the first time in a dose-dependent manner. This protection was reflected biochemically by the significant improvement in serum levels of ALT, AST, ALP and GGT, indicating the ability of *N. alba* AEE to protect hepatocytes against the deleterious effects of CCl_4_. Furthermore, the significant decrease in the serum level of bilirubin indicated that bilirubin was taken up into the liver as a function of a healthy hepatocyte. The hepatoprotective effect of the extract against CCl_4_-intoxication was further supported by histopathological examinations which showed considerable improvement of the histopathological features of the liver with *N. alba* treatment.

Silymarin is a unique flavonoid complex that has been reported to possess strong hepatoprotective properties and commonly used in experiments as a reference hepatoprotective substance [47]. Silymarin has a broad array of in vitro and in vivo activities such as anti-inflammatory, anti-apoptotic and antioxidant [48]. Our results showed that silymarin protects against CCl_4_-induced hepatotoxicity as reflected by the significant improvement in the liver enzymes and bilirubin as well as enhancement of the histopathological features of the liver which was in agreement to previous studies [46, 47]. The protective effect of a high dose of *N. alba* (200 mg/kg) against hepatotoxicity is comparable with the effect observed with silymarin (100 mg/kg) which indicates a strong hepatoprotective property of the high dose of *N. alba*.

Caspase-3 is a protein that plays a vital role in apoptosis [49]. In the present study caspase-3 was extensively expressed in the liver excised from CCl_4_-intoxicated rats denoting the correlation between CCl_4_ induced hepatotoxicity and the high level of apoptosis of the hepatocytes as previously reported [50]. *N. alba* decreased the level of caspase-3 expression while the effect of the high dose of *N. alba* is similar to that of silymarin as both drugs showed negative staining for caspase-3. Consequently, the protective effect of *N. alba* extract against CCl_4_ is mediated, in part, by inhibition of apoptosis through caspase-3 dependant pathway.

Oxidative stress has been shown to play a pivotal role in liver injury induced by CCl_4_ [51, 52]. Our results showed an obvious disturbance in oxidant-antioxidant balance of the liver subjected to CCl_4_ where injection of CCl_4_ increased the degree of lipid peroxidation as indicated by the significant increase in MDA level in the liver homogenate. Both non-enzymatic and enzymatic antioxidant defence mechanisms were deteriorated in CCl_4_-injected group. The oxidant-antioxidant status of the liver excised from CCl_4_-intoxicated rats was significantly improved by treatment with *N. alba* in a dose-dependent manner. These findings imply a profound in vivo antioxidant effect of *N. alba*. These results are consistent with the studies documented by Khan and Sultana, [12, 13], who reported that *N. alba* extract suppresses chemically-induced oxidative stress and kidney damage in Wistar rats.

The strong antioxidant activity of silymarin has been documented previously in several studies [47, 48]. In the present study the antioxidant activity of the high dose of *N. alba* is comparable to the antioxidant activity of silymarin.

TNF-α is an important inflammatory mediator that has been shown to be involved in diverse pathological processes and in our study, TNF-α is elevated significantly in the CCl_4_-intoxicated group, which was previously reported [51, 52]. Treatment with *N. alba* resulted in a significant decrease in the hepatic content of TNF-α, which is comparable, in its high dose, with silymarin. This result indicates a profound anti-inflammatory effect of *N. alba* which was in agreement with that reported in models of acetic acid-induced vascular permeability and cotton pellet-induced granuloma. In both models, *N. alba* exhibited an anti-inflammatory effect in a dose-dependent manner, which can be comparable with that of diclofenac sodium [6].

## Conclusion

The results highlight the high phenolic content of *N. alba* leaves, denoting the predominance of hydrolysable tannins, mainly ellagitannins, in addition to the flavonoid content of major antioxidant activity. *N. alba* also appeared as a rich source of essential fatty acid with high nutritional value. Administration of *N. alba* extract remarkably protected against CCl_4_-induced hepatotoxicity to an extent comparable with silymarin. The suppression of oxidative stress and the inhibition of a crucial pro-inflammatory mediator such as TNF-α might be the possible mechanisms for the hepatoprotective activity of *N. alba* that help in restoration of the physiological and histological features of the liver. This preclinical study provides convincing evidence that *N. alba* extract can control inflammatory and oxidative stress-related liver diseases.

## Abbreviations

A: Absorbance
AAE: Aqueous ethanolic extract
ALP: Alkaline phosphatase
ALT: Alanine aminotransferase
AST: Aspartate aminotransferase
CAT: Catalase
DPPH: 1, 1-Diphenyl-2-picrylhydrazyl
EFAs: Essential fatty acids
FAME: Fatty acid methyl ester
GGT: Gamma glutamyl transpeptidase
GLC: Gas Liquid Chromatography
GSH: Reduced glutathione
HR-ESI-MS/MS: High-resolution electrospray ionisation mass spectrometry
MDA: Malondialdehyde
MUFA: Monounsaturated fatty acids
PUFA: Polyunsaturated fatty acids
SFA: Saturated fatty acids
SOD: Superoxide dismutase
TAC: Total antioxidant capacity
TNF-α: Tumour necrosis factor
USM: Unsaponifiable matter
